# Effects of Brief Mindfulness Interventions on the Interference Induced by Experimental Heat Pain on Cognition in Healthy Individuals

**DOI:** 10.3389/fpain.2021.673027

**Published:** 2021-06-02

**Authors:** Louis-Nascan Gill, Vanessa Tabry, Véronique Taylor, Maxime Lussier, Kristina Martinu, Louis Bherer, Mathieu Roy, Pierre Rainville

**Affiliations:** ^1^Department of Psychology, Université de Montréal, Montreal, QC, Canada; ^2^Department of Psychology, McGill University, Montreal, QC, Canada; ^3^Centre de Recherche de l'Institut Universitaire de Gériatrie de Montréal, Montreal, QC, Canada; ^4^Department of Medicine, Université de Montréal, Montreal, QC, Canada; ^5^Center for Research, Montreal Heart Institute, Montreal, QC, Canada; ^6^Department of Stomatology, Université de Montréal, Montreal, QC, Canada

**Keywords:** pain interference, cognition, meditation, analgesia, experimental study, mindfulness

## Abstract

**Background:** Pain captures attention and interferes with competing tasks demanding cognitive effort. Brief mindfulness interventions involving both conceptual learning and meditation exercises have been shown to improve attention and reduce pain sensitivity, and could potentially reduce pain interference. This study assesses the effect of a 5-day mindfulness intervention (20 min/day) on the interference produced by thermal pain on working memory performance using a 2-back task.

**Methods:** Healthy participants were randomized into three groups exposed to mindfulness meditation training (*n* = 15), an active educational control intervention comprising only conceptual information on mindfulness (*n* = 15), or no intervention (*n* = 15). The two active interventions were administered in a dual-blind fashion and outcomes were assessed by research personnel blind to this allocation. Evaluation sessions were conducted before and after the interventions to assess the effect of pain on 2-back performance (pain interference). Importantly, both pain stimuli and the 2-back task were calibrated individually and in each session before assessing pain interference, thereby controlling for possible changes in baseline pain sensitivity and cognitive performance. Secondary outcomes included heat pain sensitivity, cold pain tolerance, cognitive inhibition, cognitive flexibility, and divided attention.

**Results:** Manipulation checks confirmed that heat pain interferes with the performance of the working-memory task. Compared to the no-intervention control group, pain interference was significantly reduced following the conceptual intervention but not the meditation intervention, although a corollary analysis suggests the effect might be due to regression toward the mean caused by baseline imbalance in pain interference. Secondary outcomes also suggested an increase in pain tolerance in the conceptual learning group only.

**Discussion:** A short mindfulness meditation intervention was insufficient to reduce pain interference but conceptual learning about mindfulness produced some unexpected benefits. Although the generalization of experimental findings to clinical pain conditions may be premature, these results highlight the importance of distinguishing the contribution of mindfulness education and meditation training in future studies. Understanding the effects of mindfulness training on pain regulation and management must take into consideration the multiple factors underlying this complex intervention.

## Introduction

Pain disrupts attention and interferes with cognitive processing (1). Pain symptoms in chronic pain patients thereby limit the ability to complete cognitively demanding tasks, imposing functional limitations beyond physical disability (2, 3). In experimental studies, pain interference on cognitive function has been suggested to be contingent on the cognitive task and the properties of the painful stimulus: interference seems to be more consistently found with divided attention or working memory tasks (4). Individual psychological factors affecting the magnitude of interference have also been identified, such as perception of pain-related threat (5), expectation of pain interference (6) and pain catastrophizing (7). Such psychological factors are examples of secondary affect of pain processing: an affective reaction to painful stimuli that is dependent upon the cognitive interpretation of the broader meaning of pain signals (8). Hence, psychological interventions that modulate the affective and cognitive dimensions of pain could potentially reduce pain disruption of cognitive processes.

Mindfulness-based interventions aim to increase present moment awareness and acceptance while reducing secondary elaborations associated with pain suffering (9). While the efficacy of mindfulness for chronic pain symptoms is not clearly established (10, 11), there is clinical evidence that mindfulness reduces pain interference of daily life tasks (12). Trait mindfulness was also found to negatively moderate the relation between pain intensity and pain interference in a cross-sectional study of cancer survivors suffering from chronic neuropathic pain (13). Consistent with these clinical findings, we recently observed a reduction in the impact of experimental heat pain on working-memory performance in healthy individuals with high trait mindfulness (14).

Interestingly, brief mindfulness intervention of 4 daily sessions of 20 min of attention monitoring meditation may be sufficient to induce change in acute pain perception (15–17), although such results are not systematically reproduced (15, 18). Brief mindfulness interventions are therefore a relevant experimental paradigm, but more research is warranted regarding this nascent form of mindfulness intervention to better define the contributing factors and the relevant outcomes.

Here, we hypothesized that a short meditation intervention involving mindfulness attention monitoring would reduce the disruptive effect of acute pain on cognitive performance. The experimental model and the primary outcome were defined based on our previous study (14). To further circumscribe the effect of meditation training, the target intervention group was compared to an education group exposed to 5 daily sessions involving only conceptual information about mindfulness, and to a no-intervention control group. We also assessed baseline pain sensitivity, pain tolerance as well as cognitive inhibition and divided attention capacities, to document possible associations between changes in pain regulation and other executive functions (19).

## Materials and Methods

### Study Design

This experimental study adopted a mixed design to test the reduction (post- vs. pre-intervention) of the interference produced by pain on cognitive performance (pain interference) following a 5-days mindfulness meditation training (Meditation Group), compared to an active control group receiving conceptual education about mindfulness (Conceptual Group), and a passive control group with no intervention (Control Group). All interventions and testing took place at the Research Centre of the Institut Universitaire de Gériatrie de Montréal (CRIUGM, Canada). All procedures conformed to the standards set by the latest revision of the Declaration of Helsinki and were approved by the ethic committee of the CRIUGM (CER-IUGM 15-16-11).

### Participants and General Procedure

Healthy adults were recruited from the University de Montréal campus and the general community, through social network groups (e.g., the Facebook group of student associations) and local classified ad websites. Forty-five volunteers were recruited on the basis of previously published criteria about healthy participants in pain studies (20). Exclusion criteria included chronic pain, neurological or psychological/psychiatric disorder; knowledge of, or experience with, meditation, yoga, or mindfulness; taking any medication or psychoactive drugs; hypertension or Raynaud's syndrome (see http://links.lww.com/PAIN/A101; participants filled an online French translation of the questionnaires and checklist). Additionally, the online form also included the French version of the Beck Depression Inventory [BDI - (21)] as well as specific questions to assess meditation experience and prior knowledge about mindfulness.

Given the absence of literature on short interventions to reduce pain interference, power analysis was conducted based on a controlled study showing a significant decrease in pain sensitivity following a short mindfulness intervention (16). We assumed a very conservative estimate of test-retest reliability of pain rating of 0.50 (22). The analysis was conducted on G-Power (23), assuming sphericity, with an α error of 0.05 and power of 0.80. The total sample size was 30 (10 per groups). A total sample size of 45 (15 per group) was then determined as a realistic sample size to detect a possible reduction in pain interference by a brief mindfulness intervention in a first experimental study.

Once eligibility was confirmed, participants were allocated to one of the three groups (1:1:1) until 15 participants per group, with a random list generated on GraphPad software (http://www.graphpad.com/quickcalcs/randomize1/). The list was generated by the principal investigator (the first author), who was responsible for the enrollment, randomization, group assignment, and training sessions for both the mindfulness Meditation Group and the control Conceptual Group. Subjects were only told that they were assigned to either a mindfulness intervention involving five training sessions or a no-intervention group. Subjects of the two intervention groups were given no further details about the comparison of the two different mindfulness interventions. To avoid confusion with double-blinding applied in pharmacological clinical trials, this design is referred to as “dual-blind” and is recommended for psychological intervention such as mindfulness training (24).

Importantly, the recruitment ads and the information provided with the consent form indicated that the goal of the study was to investigate the effect of a mindfulness intervention on pain and cognition. To avoid inducing specific expectations about the main hypothesis, the main outcome of the study (pain interference) was not specified.

**Figure 1:**
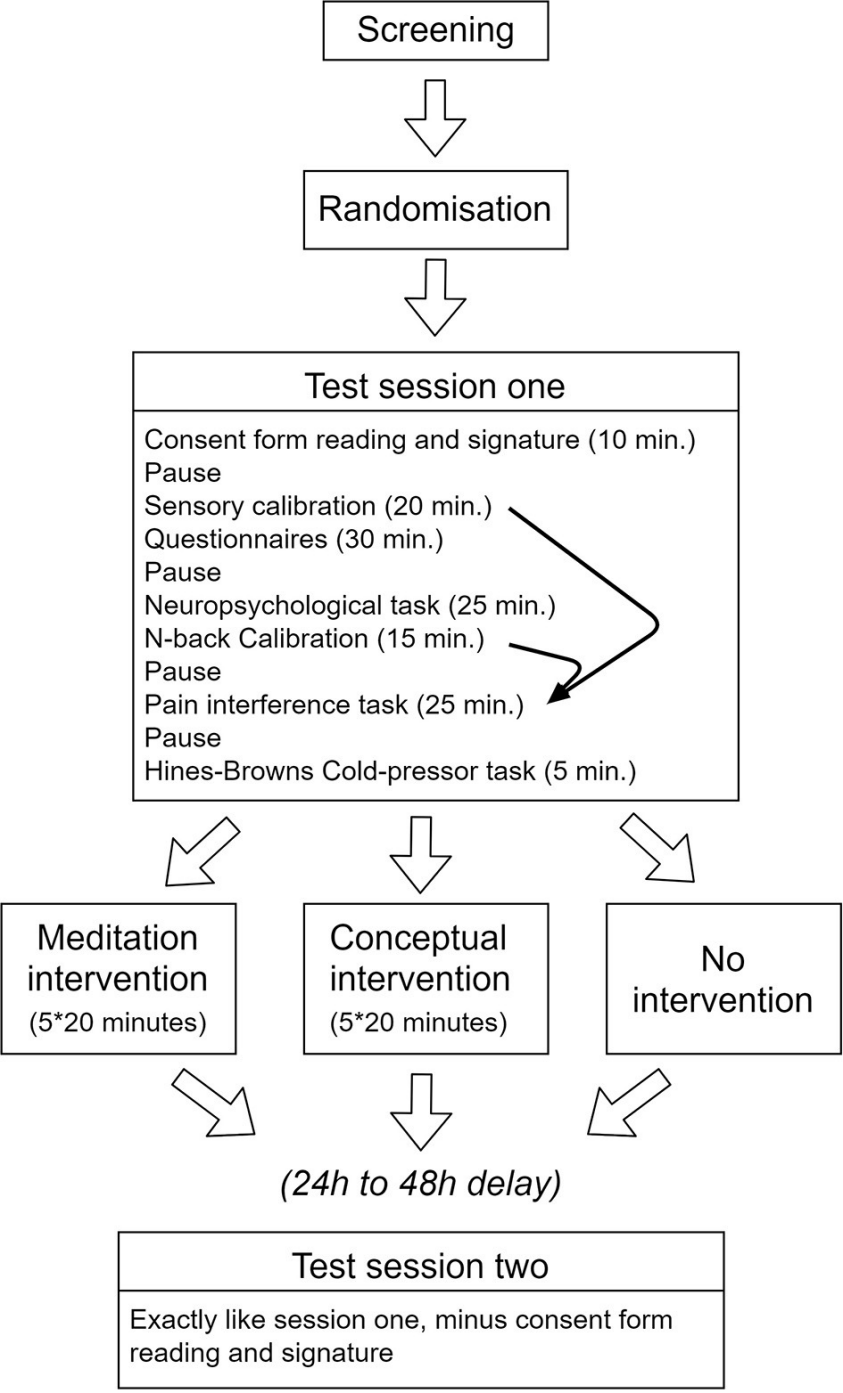
Schematic illustration of the procedures. Note that individual results of the sensory calibration and the N-back calibration procedures were used to determine the parameters used in the target experiment assessing pain interference (curved arrows). All other tests and questionnaires were completed to describe the samples and control for potential confounding changes in pain and in cognitive performance.

Within 4 days of this first testing session, participants in the Meditation and Conceptual intervention began the 5-day mindfulness intervention (see below in the section “interventions”). The post-intervention testing was then completed within 2 days of the last intervention session. The participants in the passive Control group completed the second testing session after a similar delay (8–12 days) following the first session. Participants of all three groups were asked to maintain a regular lifestyle, including sleep habits and caffeine intake throughout the study and received monetary compensation for their participation. Figure 1 illustrates the overall study procedures.

Two female experimenters conducted the testing sessions and were blind to the group assignation of the participants to the Meditation or Conceptual Group. All subjects were tested twice by the same experimenter. However, blinding of experimenters could not be insured for the no-intervention Control Group in the second testing session due to several logistic constraints and slightly different instructions given by the experimenter in the pauses before each test (see below).

### Interventions

Both the meditation intervention as well as the conceptual intervention were delivered by the first author (LNG), a graduate student in psychology with 10 years of mindfulness meditation experience (>1,000 h of practice and two 10-day retreats in a Vipassana center), who received a mindfulness intervention training recognized by the professional association of Québec's psychologists (Ordre des Psychologues du Québec - OPQ). This training was provided by the Mindspace clinic and involved the development of mindfulness scripts under expert supervision (https://www.mindspacewell-being.com/programs/introduction-to-mindfulness-for-psychotherapists/). Both interventions were delivered in five one-on-one daily sessions of 20 min.

#### Meditation Intervention

Preparation phase (2 min): In a quiet room with dim lighting, participants were asked to sit on a Zen cushion (“zafu”) in a position that they found comfortable and in which their spine would be straight. They had about 1 min to try different seating positions. Thereupon, they were asked to close their eyes, try to keep their body still and listen to the instructions. The instructor encouraged the participants to let go of any other preoccupations and to fully focus their attention on the exercise.

Meditation phase (16 min): Over the course of the intervention, three different meditation exercises were practiced, exposing participants to different styles of both focused attention and open monitoring, two fundamental aspects of mindfulness attention regulation meditation (25, 26). These exercises involved (1) focused breathing, (2) body scan and (3) labeling exercises and were initiated with guidance from the instructor followed by silent periods. The full instructions are available in the Supplementary Material.

Feedback phase (2 min): In the last period of every session, participants were invited to share their experience with the instructor. The goal of this phase was to make sure that the participant understood the exercises.

#### Conceptual Intervention (Active Control)

Preparation phase (2 min): The same room and the same seating instructions as the meditation intervention were used for the conceptual intervention. However, participants were not instructed to close their eyes and were simply asked to concentrate fully on what the instructor was saying. They were also encouraged to ask questions during the session.

Education phase (18 min): Slightly modified excerpts from the book “Wherever you go, there you are: Mindfulness meditation in everyday life” (27) were read to the participants. The word “meditation” was never mentioned. When it was possible to do so, it was removed and replaced with the term “mindfulness.” If such replacement affected the meaning of the text, different excerpts were chosen. None of the excerpts selected included a description of formal mindfulness exercises, although some informal exercises were described, e.g., suggesting paying attention to physical sensation during moments of mind wandering.

Three times per session, the instructor elicited discussion, by asking questions to the participant, e.g.,: do you agree with the author?, Have you ever realized that your mind sometimes wanders when you are doing certain tasks?, Do you think the state of mind that is being described could be useful to you? A description of the content of each session is provided in Supplementary Material.

#### Control (No Intervention)

Participants in the control group did not receive any intervention.

### Testing Procedures and Measures

The general procedure of the pre- and post-intervention assessments of pain sensitivity, cognitive function and pain interference on cognitive performance is described in Figure 1.

#### Pain Interference Assessment

The notion of pain interference is frequently used in clinical literature to refer to any limitation (e.g., movement) imposed by the chronic pain condition. Here the term specifically refers to the decrease in working-memory performance caused by experimental heat pain. The pain interference task is detailed below and is also further described in our previous study (14).

##### Sensory Calibration

The temperature used for the painful and non-painful stimulation in the interference task was calibrated for each participant to control for inter-individual differences in pain sensitivity (4, 14, 28). This procedure also generated two measures of pain sensitivity: pain threshold and temperature required to produce a moderately painful experience.

Thermal stimulation was delivered on 4 different areas on the surface of the non-dominant forearm with a Medoc Thermode contact probe (TSA Neuro-sensory analyzer, Medoc Ltd. Advanced Medical System, Israel). In total, 28 stimulations of seven different temperatures (40, 44, 45, 46, 47, 48, 49°C) were administered in a random order (method of constant stimuli). Each stimulation involved 2.5 s of increase from the 32-degree baseline temperature, 8 s of plateau at the target temperature and 2.5 s of decrease to baseline.

Instructions and visual analog rating scales (VAS) were presented on a monitor in front of the participant using E-Prime2 Professional (Psychology Software Tools, Sharpsburg, PA). After each stimulus, participants rated the sensation as painful or not painful, with a mouse click with the right hand. If the stimulation was categorized as “not painful,” participants then rated the warmth of the sensation on VAS, going from 0 (no warmth at all) to 100 (very warm, without pain). If the sensation was rated as “painful,” participants then rated pain intensity (0 – “not intense at all” to 100 - “extremely intense”).

Warmth and pain evaluations were then plotted on a single scale going from 0 to 200. An exponential stimulus-response curve was generated for each participant to interpolate the pain threshold (100/200 on the scale), as well as a moderately painful temperature (140/200). In addition, a warm temperature (70/200) was also determined to use as the control temperature in the interference task (see below). In other words, the temperature of the painful and warm stimulation was individualized for each participant and for each testing session, as to match a perceived pain intensity of 40/100, or a warm sensation of 70/100. Those specific pain intensity/warmth levels were chosen because our previous study (14) showed that they were adequate to produce pain interference. More specifically, a painful stimulation that is associated with a 40/100 pain intensity rating is sufficient to produce a decrease in 2-back performance (see below), but not too intense as to cause participants to abandon the experiment when repeatedly administrated. A 70/100 warmth stimulation is used as a control stimulation to ensure the interference effect is related to pain and not simply thermal sensation.

##### Two-Back Calibration

The N-back is a continuous performance task measuring working memory performance (29). A series of individual letters appeared successively on a screen for 500 ms each, preceded by a 250 ms fixation cross and a blank inter-stimulus interval adjusted (calibrated) individually to attain comparable performance across subjects. Participants indicated if the letter being presented (the possible letters were C, F, J, N, Q, S, V, and X) was the same or different from the “nth” previous letter. A 2-back task was used, so that each letter had to be compared with the letter presented two letters back. The task was administered with a computer on E-Prime2 Professional (Psychology Software Tools, Sharpsburg, PA). Participants used the computer mouse to indicate if the letter was the same (left click) or different (right-click) from the letter presented two letters back.

Performance accuracy was calculated with the A-sensitive index statistic, a nonparametric estimate of sensitivity (30). The goal of the calibration task was to familiarize participants with the task and to determine task parameters to obtain a performance of 0.75 < A < 0.85. This was done using an adaptive procedure by manipulating the inter-stimulus interval by adjusting the duration of the blank interval (mask) between successive letters: longer intervals are easier, while shorter intervals are more difficult (14). By the end of the calibration task, a mask duration that reliably produced a performance of 0.75 < A < 0.85 was determined and subsequently used in the interference task. Therefore, the difficulty of the 2-back was also adjusted for each participant at each session to control individual differences and possible practice effects on working-memory performance.

##### Pain Interference Task

**Figure 2:**
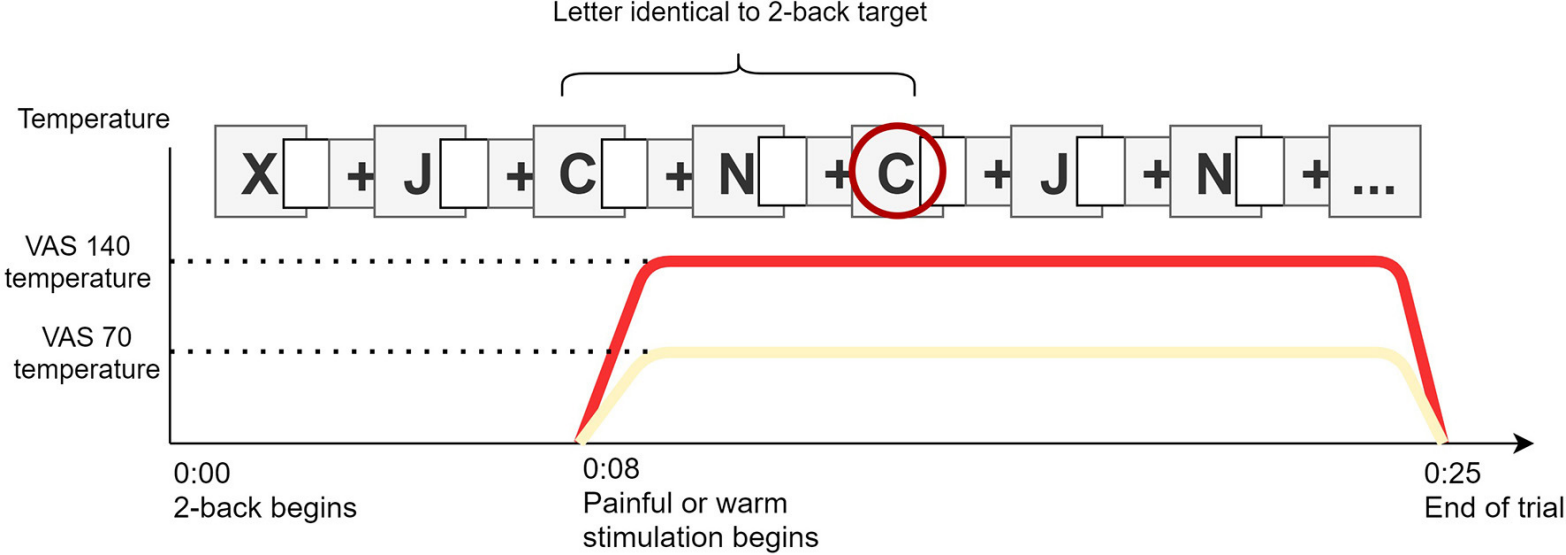
Illustration of the pain interference task. Painful (VAS140) or warm (VAS70) stimulation starts 8 s after the beginning of the 2-back. With squares illustrate the calibrated blank inter-stimuli interval. Letter are presented until the trial ends.

During the pain interference task, both the intensity of thermal stimulations and task difficulty were controlled based on the results of the calibration phase (28). To assess cognitive interference caused by pain, participants completed 18 series of the 2-back task while they received thermal stimulation on the left forearm, as shown in Figure 2 (note that each 2-back series is defined here as one trial). The number of n-back items administered in a trial was adjusted according to the calibration results to keep the duration of a trial constant at 25 s. The thermode was applied to a different spot of skin on each trial. Half of the stimulations were set to produce moderate pain (VAS140) or non-painful warmth (VAS70) according to the calibration procedure, delivered in random order. On each trial, the thermal stimulus started 8 s after the beginning of the 2-back task and the thermal plateau ended 17 s later, with the last 2-back item. The pain interference score (**PIS**) was calculated as the difference in A-sensitivity index to the 2-back trials between the painful and non-painful conditions.

#### Cold Pressor Test

The Hines-brown cold pressor test was performed as a secondary pain tolerance task to document potential changes in the affective dimension of pain that might be missed by the heat pain sensitivity test (31). Participants immersed their left hand in circulating water at 4.5°C for the longest period they could tolerate, for a maximum duration of 300 s (maximum duration unknown to the participant). When the participants retracted their hand, they rated pain intensity and pain unpleasantness between 0-no pain/not unpleasant at all, and 100-extremely intense pain/extremely unpleasant (32).

#### Neurocognitive Tasks

Corollary measures of cognitive inhibition and divided attention were collected (without pain interference) pre- and post-intervention to explore possible mediators of the hypothesized changes induced by the mindfulness meditation on pain interference. However, the main results did not justify testing mediation models and the description of these tasks is relegated to the Supplementary Material.

#### Questionnaires

Questionnaires were used to document possible group differences in mindfulness and some psychological factors that may affect pain perception and pain interference. Participants filled Five Factor Mindfulness Questionnaire (FFMQ) and Mindful Awareness Attention Scale (MAAS) (33–36); State-Trait Anxiety Questionnaire (STAI) (37, 38), Pain Catastrophizing Scale (PCS) (39, 40) and Pittsburgh Sleep Quality Index (PSQI) (41, 42). Participants could fill the questionnaires in French or in English.

All questionnaires were administered at both testing sessions except for the PSQI and the Trait Anxiety Questionnaire, administered only once before the first testing session. There were no statistically significant changes in questionnaires score between sessions one and two. Only session 1 (pre-test) scores are reported (session 2 scores, as well as test-retest correlations, are presented in (Supplementary Tables 1, 2).

#### A Priori Expectation of Improvement

The effects of expectations on pain and cognitive performance are well documented (43–47). At the beginning of the first intervention session, a quick description of the intervention was provided to the participants, and they were asked to rate their expectations (5-point Likert scales) concerning the effectiveness of the intervention on pain and cognitive performance. At the end of the first testing session, participants of the control group also rated their expectations of changes in pain and cognitive performance from the first to the second testing session. Participants were not asked to rate their expectations regarding pain interference to avoid directing their attention to the primary outcome of the study.

#### Pauses

We scheduled pauses of 2 min between tests (see Figure 1). In session one, participants were simply asked to sit, close their eyes, and wait for 2 min. In session 2, participants were asked to sit, close their eyes and use the time to remember what they learned during their intervention. Participants from the control group received the same instruction as the first session. The experimenter left the room at this moment so that participants would not feel observed or judged and to reduce the risk of un-blinding the experimenter to the Meditation Group vs. Conceptual Group.

### Statistical Analysis

Data extraction from E-Prime output files was conducted using Matlab® version 7 (MathWorks, Natick, MA, USA), and statistical analyses were conducted using IBM SPSS statistic (version 23), with the exception of Bayesian analysis of the null hypothesis (48, 49). All dependent variables were checked for extreme scores corresponding to an absolute z-score of 3.29 or more (50).

The main hypothesis that pain interference would decrease after the mindfulness meditation intervention was tested by comparing the PIS observed in the second session to the PIS obtained in the first session across the three groups, using a mixed-model ANOVA of GROUP (Meditation, Conceptual, Control) x SESSION (Pre vs. Post), with Tukey' *post-hoc* test. Change scores (pre-to-post intervention) were also compared between groups using an unplanned ANCOVA controlling for individual differences in baseline PIS.

Effects of the intervention on pain sensitivity and cognition were also assessed with mixed-model GROUP x SESSION ANOVA's. Mean tolerance times in the Hines-Brown Cold pressor task was computed in each group and each session, but the distributions showed important deviation from normality (bimodal distribution), precluding parametric comparisons (51). Log-rank test on Kaplan-Mehier curves, a nonparametric survival analysis, was used (52). Such analysis allows comparison of the probability of an event to occur through time and across different groups, in presence of censored observation. In our case, the target event was hand retraction from the cold pressor and a censored observation referred to a trial where the participant would keep his hand submerged for the complete duration of the test (300 s). This test was performed to compare groups on duration of hand immersion at session 1 and 2. In addition, a mixed-measure GROUP X SESSION ANCOVA was performed on pain intensity and unpleasantness ratings of the cold-pressor test using immersion duration as a covariate.

When testing the null hypothesis was of interest, or when statistical significance was near *p* = 0.05, Bayesian statistics were also performed (48, 49). Bayesian analyses were conducted with Dienes online calculator on group mean differences between the pre- vs. post-intervention measurements [https://medstats.github.io/bayesfactor.html, (48)]. The predictions of the alternative hypothesis were modeled with a normal two-tailed distribution centered on zero (theoretical mean = 0). Theoretical standard deviations were derived from a study on mindfulness and pain (16) for pain-related variables, and from a study on manipulation of pain expectation for the expectations analyses (53). Interpretation of Bayes factors followed the recommendation of (54). It is worth noting that, while Bayesian statistics produce a continuous qualification of evidence (as opposed to the alpha threshold of the null hypothesis significance testing approach), a Bayes factor above 3 roughly corresponds to the level of evidence “for H1” provided by the rejection of H0 when *p* < 0.05. Conversely, a Bayes factor under 1/3 implies the same level of evidence, but for H0; Bayes factors between 1/3 and 3 are interpreted as “anecdotal” or “inconclusive” (55).

## Results

### Sample Description

**Figure 3:**
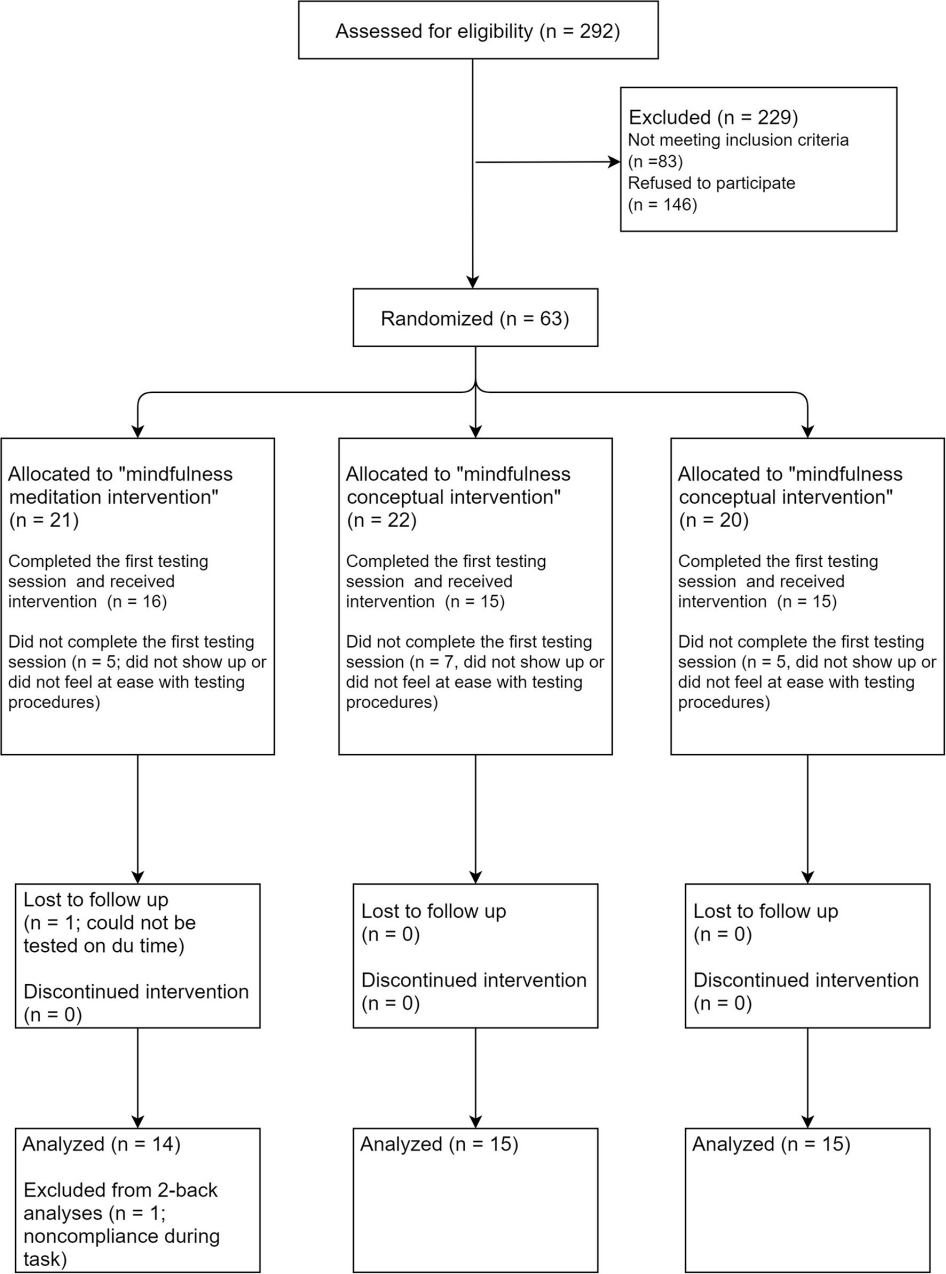
Consort-like flowchart of the participants.

The selection of participants and final sample are described in Table 1 and Figure 3. A total of 292 potential volunteers contacted our research team to inquire about the study and 63 were recruited and allocated to one of the three groups. Refusal to participate and attrition were mainly due to the number of visits required for the study and difficulty in scheduling all the sessions within the time frame prescribed by the study design. From the 63 participants enrolled, 15 did not show up to the first testing session and three stopped the experimentation at the calibration phase because they felt uncomfortable with the testing procedure. A total of 45 participants had a complete, or almost complete, data set (*n* = 15 per group) and entered the final analysis. There was no difference in the ratio of male/female per group: χ ^2^ (2) = 1.25, *p* = 0.54.

**Table 1:** Demographic data and a priori expectations (mean with standard deviations in parenthesis).

**Group**	**Meditation**	**Conceptual learning**	**Control**
	**(*n* = 15)**	**(*n* = 15)**	**(*n* = 15)**
Female: male	6:9	9:6	8:7
Age (y.)	24.9 (3.9)	23.7 (4.4)	25.5 (4.9)
Education (y.)	16.8 (2.6)	15.1 (2.3)	16.7 (2.6)
MAAS	55.47 (9.5)	61.7 (13.2)	58 (12.6)
FMMQ - Observe	20.87 (4.5)	20.1 (4.0)	21.7 (2.6)
FFMQ - Awarness	21.73 (2.3)	22.2 (3.7)	21.6 (3.6)
FFMQ - Describe	23.87 (5.1)	24.8 (3.8)	22.7 (3.4)
FFMQ - Nonreact	25.93 (3.5)	28.6 (1.9)	26.7 (3.4)
FFMQ - Nonjudge	22.60 (3.20)	22.2 (3.6)	22.1 (3.1)
PCS - Rumination	8.13 (3.89)	7.3 (4.9)	6.8 (4.1)
PCS - Magnification	3.73 (2.63)	2.9 (2.2)	3.8 (2.8)
PCS - Helplessness	5.93 (3.95)	6.1 (5.6)	6.27 (3.77)
PSQI	5.47 (2.33)	4.6 (1.6)	2.67 (1.6)
STAI - Trait	37.4 (7.44)	32.3 (9.7)	32.2 (9.6)
Expectation on pain	3.3 (0.80)	3.5 (1.0)	3.0 (0.80)
Expectation on cognition	3.6 (0.90)	3.2 (1.0)	3.4 (1.4)

There was no significant GROUP differences on any questionnaire (all p's > 0.1) and no significant SESSION effect or GROUP X SESSION interaction on the questionnaires filled at both sessions (all p's > 0.05).

### Pain Interference (*Primary Outcome*)

#### Sensory Calibration

Pain sensitivity decreased slightly but significantly (*p* < 0.05) between sessions on VAS70 and VAS140 (i.e., increased temperature to produce the same subjective level of pain). There was no interaction with the GROUP (all p's > 0.75), consistent with a non-specific decrease in pain sensitivity independent from the intervention. Detailed statistical results are provided in Supplementary Table 3.

#### 2-Back Calibration

The mask duration increased significantly from the first to the second testing session across all groups to control for baseline task performance in the calibration phase (*p* < 0.05). There was no significant interaction GROUP X SESSION (*p* = 0.9). This is consistent with a non-specific training effect across all groups. Also, test-retest correlations (*r* = 0.78, *p* < 0.001) are consistent with previous literature on n-back performance (56) and suggests an acceptable test-retest reliability. Detailed statistical results of the calibration procedure are provided in Supplementary Table 4.

#### Pain Interference Task

The 2-back performance at Session 1 confirmed that performance decreased in the VAS140 (painful) condition compared to the VAS70 (warm) condition (*p* < 001, *d* = 0.62). Mean performance was 82,35% in the painful condition (CI = 79,92 to 84,78%) and 86, 55% in the warmth condition (CI = 84,27 to 88,84%). This indicates that the task worked as intended and that pain interference was produced.

**Figure 4:**
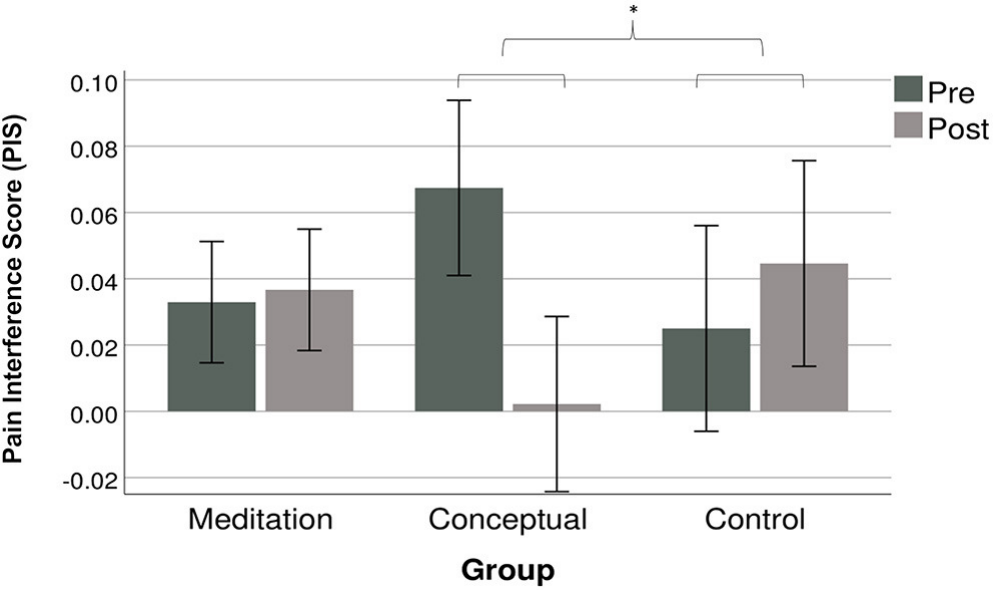
PIS mean (95% within subjects CI's) for each group at both testing session. The PIS is the difference between the A-sensitivity index of the 2-back task in the warm condition minus the pain condition. A higher PIS indicates more pain interference. *p = 0.017.

Pain Interference Scores (PIS), assessed using the difference in 2-back performance (A-sensitivity index) between painful and warm condition, are shown in Figure 4, with within subject 95% CIs (57). The analysis revealed no main effect of SESSION [*F*(1,41) = 0.99, *p* = 0.3, η^2^ = 0.02] or GROUP [*F*(2,41) = 3.26, *p* = 0.7, η^2^ = 0.02]. However, PIS showed a significant interaction GROUP X SESSION [*F*(2,41) = 3,48, *p* = 0.040, η^2^ = 0.15]. *Post-hoc* analysis in one-way ANOVA on change scores between sessions revealed no significant difference between the PIS change between the Meditation Group and the Control Group (*p* = 0.66). However, PIS changes in the Conceptual Group were significantly different from those observed in the Control Group (*p* = 0.017, *g* = 0.85), suggesting a decrease in pain interference following the conceptual intervention. This effect in the Conceptual Group was not statistically significant when compared to the change in PIS observed in the Meditation Group (*p* = 0.051, *g* = 0.86).

The Bayes factor for the comparison between PIS change in the Meditation and Control Group was 0.54, indicating anecdotal evidence in favor of the null hypothesis. This indicates that the data is inconclusive regarding the difference PIS changes observed in the Meditation and Control Group. Bayes factor for the comparison between the Meditation and Conceptual Group was 18.23, suggesting strong evidence in favor of the alternative hypothesis (difference between the PIS change in these two groups). The Bayes factor for the comparison between PIS change in the Conceptual and Control Group was 42.89, suggesting very strong evidence in favor of the alternative hypothesis, consistent with a larger effect of the intervention in the Conceptual Group.

Given the higher baseline PIS of the Conceptual Group, the significant improvement may be caused by a regression toward the mean. That is, higher baseline values due to chance are more likely to decrease following the intervention (58). To control for a potential regression toward the mean, an unplanned ANCOVA was conducted on PIS changes across measurement time. The effect of the intervention was not significant after controlling for individual differences in PIS levels at baseline [*F*(2, 41) = 2.07, *p* = 0.14, η^2^ = 0.094].

### Cold Pressor (*Secondary Outcome*)

Pain tolerance (hand immersion time), proportion of subjects tolerating the maximum duration of the test, and pain intensity and unpleasantness ratings, are reported in Table 2. The larger increase in mean immersion time, and in the proportion of participants doing the test for the full duration, is found in the Control Group. Large CI's reveal important variability in pain tolerance and Log-Rank comparisons of Kaplan-Mehier survival curves at session one or two showed no difference on hand retraction probability between the three groups (session 1: χ2 = 1.20; *p* = 0.52; session 2: χ2 = 2.21; *p* = 0.30). Overall, these results do not show evidence of an effect of the intervention on pain tolerance. Distribution of the hand immersion time and survival curves are provided in the (Supplementary Figure 2).

**Table 2:** Hand immersion time and pain evaluation of the hines-brown cold-pressor task (mean with 95% CI).

**Group**	**Meditation**	**Conceptual learning**	**Control**
	**(*n* = 15)**	**(*n* = 15)**	**(*n* = 15)**
Immersion time. session 1 (in seconds)	116.9 (52.1 to 181.8)	156.5 (84.9 to 228.2)	79.6 (36.6 to 122.6)
Immersion time. session 2 (in seconds)	96.5 (37.1 to 156)	164.4 (97.4 to 231.4)	122.1 (53.2 to 191.00)
Pain intensity, session 1 (on a 0 to 100 scale)	77.9 (67.4 to 88.3)	80.0 (72.0 to 88.0)	84.4 (76.5 to 92.3)
Pain intensity, session 2 (on a 0 to 100 scale)	76.4 (63.5 to 89.3)	70.0 (59.5 to 80.5)	84.1 (77.5 to 90.8)
Pain unpleasantness session 1 (on a 0 to 100 scale)	71.2 (56.3 to 86.1)	79.7 (69.8 to 89.6)	87.5 (80.4 to 94.5)
Pain unpleasantness session 2 (on a 0 to 100 scale)	69.3 (53.5 to 85.1)	66.0 (52.2 to 79.8)	88.9 (82.5 to 95.3)

Cold pain ratings (intensity; unpleasantness) decreased on the second testing session but this main effect did not meet the significance threshold when controlling for the tolerance time [ANCOVA, Intensity: *F*(1,41) = 3.89, *p* = 0.056, η^2^ = 0.09; Unpleasantness: *F*(1.41) = 2.46, *p* = 0.057, η^2^ = 0.07]. More importantly, there was a significant interaction between GROUP and SESSION on pain intensity [*F*(2;41) = 3.85, *p* = 0.029, η^2^ = 0.19] and pain unpleasantness [*F*(2,41) = 3.83, *p* = 0.030, η^2^ = 0.16]. This indicates that the effect of sessions on cold pain varied between groups. The effects remained significant when controlling for baseline scores [Intensity F(2;41) = 3.89, *p* = 0.028, η^2^ = 0.16; Unpleasantness *F*(2;41) = 4.56, *p* = 0.016, η^2^ = 0.17].

For pain intensity, *post-hoc* analysis revealed that changes in pain between sessions in the Meditation Group did not differ from those observed in the Control Group for ratings of both pain intensity (*p* = 0.7, *g* = 0.16) and pain unpleasantness (*p* = 0.6, *g* = 0.28). However, analyses revealed a significantly larger change in the Conceptual Group when compared to the Control Group, for both pain intensity (*p* = 0.03, *g* = 0.88) and unpleasantness (*p* = 0.023, *g* = 0.78). Change in pain intensity and unpleasantness was also larger for the Conceptual Group when compared with the Meditation Group, although the difference did not reach statistical significance [pain (*p* = 0.056, *g* = 0.60); unpleasantness (*p* = 0.072, *g* = 0.55)]. These effects indicate that only the conceptual intervention reduced pain significantly during the cold pressor task. Pairwise comparisons on pain intensity or unpleasantness change over time between groups were also computed on adjusted means, as to control for the confounding effect of the immersion time covariate. The results are similar (pain intensity: Meditation vs. Conceptual learning *p* = 0.11, *g* = 0.59; Meditation vs. Control *p* = 0.29 g = 0.38, Conceptual learning vs. Control *p* = 0.01, *g* = 0.98; Pain unpleasantness: Meditation vs. Conceptual learning *p* = 0.10, *g* = 0.56; Meditation vs. Control *p* = 0.25 *g* = 0.41, Conceptual learning vs. Control *p* = 0.01, *g* = 0.98).

However, it should be noted that exploratory analyses were also conducted on cognitive and personality measures (see Supplementary Material). The interaction effect for both pain intensity and pain unpleasantness would not remain significant if adjusted for all the exploratory analyses.

### A Priori Expectation of Improvement

Participants of all three groups generally expected a reduction in pain sensitivity and some improvement in cognitive performance in session 2 (Table 1). However, expectations scores were not significantly different between groups for pain [*F*(2,41) < 1 *p* = 0.49] or cognitive performance [*F*(2,41) < 0.60; *p* = 0.60].

The Bayes factor for the pairwise comparison between pain expectations in the three groups varied between 0.07 and 0.19. These values represent moderate evidence in favor the null hypothesis. The Bayes factor for the pairwise comparison between cognitive performance expectations in the three groups varied between 0.10 and 0.27. These values represent moderate to strong evidence in favor of the null hypothesis. These results confirm an absence of group differences in expectations of the efficacy of the intervention, or test repetition in the control group, on pain or cognitive performance.

## Discussion

This study tested the main hypothesis that a short intervention involving mindfulness meditation training would reduce the interference produced by acute pain on cognitive performance. This was not confirmed as the meditation group did not improve following training compared to both the active and the no-intervention control groups. However, the active control group receiving conceptual education about mindfulness did show significant reduction in pain-induced interference. This reduction is supported by the planned ANOVA and Bayesian analysis.

Secondary outcomes suggested a significantly larger reduction in cold pain perception (controlling for immersion time) only in the conceptual group and Bayesian analysis further confirmed an improvement in this group when compared to the other two groups. No other significant changes were observed in cold pain tolerance, heat pain perception, or cognitive performance on the Stroop test, the dual task, or the 2-back task (see Supplementary Material). A priori expectations about changes in pain and cognitive performance measured before the interventions were also comparable across groups. Given the large number of statistical tests performed on secondary outcomes, the significant results in the cold pain measures should be interpreted with caution.

### Meditation Training

Results indicate that five daily sessions of mindfulness meditation based on attentional training is not sufficient to reduce pain interference. Similarly, secondary measures of pain sensitivity, pain tolerance or cognitive performance did not show improvement. Those results are observed in the context of a carefully designed meditation intervention conducted by a facilitator with notable meditation experience and a recognized mindfulness certification, as previously recommended (24). The direction of the mean pain interference score changes (increase in Meditation group and decrease in Conceptual group) indicates that insufficient power is not a satisfactory explanation for the discrepancy of the present results with previous reports suggesting improvements following meditation training.

What specific factors could explain the negative results regarding the effects of mindfulness meditation training? The comparison with studies using short mindfulness intervention that reported a positive effect on similar variables suggests that the delay between meditation practice and pain testing should be taken into account. Benefits induced by mindfulness meditation might be short-lived and temporally dependent upon the practice of meditation during or immediately before the pain test.

Previous studies have found that short attention regulation training produce stronger analgesic effects than distraction and placebo (16, 17, 59–61). However, the analgesia reported was produced in a context where participants had just practiced a guided meditation and were specifically asked to practice focused attention exercises while receiving a painful stimulation. Here, participants were not asked to meditate in the post-intervention session. They were only asked to close their eyes very briefly before each test and to remember what they had learned during the training sessions. Actively practicing a mediation exercise during pain may be necessary to produce hypoalgesic effects following short-term training. Our results suggest that a brief mindfulness training does not affect pain interference, pain tolerance or pain sensitivity tested on a separate day that did not involve active guided meditation.

Learning to engage in meditation exercises might be challenging and, in some contexts, counterintuitive (62). Engaging rapidly in meditation without the presence of a facilitator, or without specific instructions may also be too difficult for inexperienced meditators. This may be even more crucial in the context of extensive assessment as conducted in the present study, where participants may lack motivation and/or the cognitive resources to engage meaningfully and persistently in meditation.

Participants in the meditation group may even have actively tried to preserve their regulatory resources for the demanding tasks (63, 64). Another study using meditation-novice healthy participants has found that brief mindfulness meditation altered regulatory resources in the context of pain regulation (65). Novice mindfulness meditators are perhaps vulnerable to ego-depletion (66) or similar motivational limitations (67). Indeed, ego-depletion could explain why mindfulness can sometimes produce suboptimal or iatrogenic effects (68). For example, awareness of breath or body can increase the stress response of novice meditators, instead of reducing it (69). While our results do not provide direct support for the ego-depletion interpretation, it is important to stress that a feature of our methodology involved long assessment sessions. This is not the case with previous studies that showed a mindfulness-induced improvement in pain regulation (16, 17, 59–61).

### Conceptual Learning About Mindfulness

An unexpected result of the present study is the reduction of pain interference and cold pain perception following the conceptual learning intervention. This intervention was first designed as an active condition to control for non-specific effects of successive visits to the laboratory in the context of a mindfulness-based intervention (24). However, the Conceptual group was exposed to knowledge about mindfulness. The basis of mindfulness approaches is to promote an open and acceptance attitude, including toward aversive events, and conceptual education likely contributes to such benefits (9, 70, 71). In the context of very brief interventions, being exposed to, and discussing mindfulness-related concepts might be sufficient to develop curiosity about habitual behavioral, emotional and cognitive response, notably to aversive stimuli like pain, and potentially modify secondary pain affect (8), resulting in reduced pain interference.

Another possible explanation for the observed effect of the conceptual intervention relates to suggestions. Pain perception is consistently modified by suggestions (72), and similar effects have been reported on the interruptive effects of pain on cognitive performance (6). Hypnotic suggestions also take advantage of this mechanism to generate reliable pain relief (73, 74). While mindfulness was originally conceptualized as very distinct from hypnosis (e.g., a non-striving approach as opposed to the goal-oriented perspective of hypnosis), the two approaches present many similarities (75, 76). Importantly, manipulation of meditators' expectations may be a central mechanism of mindfulness interventions (77). The present results may therefore be explained by such process, as the conceptual group was more extensively exposed to mindfulness education explicitly suggesting adopting a more open and attentive attitude, and implicitly suggesting reduced pain interference (e.g., reacting with flexibility and acceptance to aversive events).

Finally, it should be noted that the decrease in interference and cold pain ratings in the Conceptual group are statistically significant only when compared to changes observed in a passive, non-blinded, control group. Moreover, mean PIS at Session 1 (pre-intervention) is higher for the conceptual learning group than for the two other groups (see Figure 4). While testing for baseline difference is not a recommended practice for randomized studies (78, 79), the diminution of PIS in the active control intervention group could be partly explained by the higher value at baseline. Higher baseline values are likely due to chance and thus likely to return to normal (i.e., lower) value at *post-test*. This phenomenon is called “regression toward the mean” and can be accounted for by adding baseline scores as a covariate (58) ANCOVAs showed that pain interference scores reduction in the conceptual group was not statistically significant when controlling for baseline interference. Conversely, cold pain perception results remained significant. Regression toward the mean likely explains the reduction in pain interference, but not in cold pain intensity and unpleasantness.

### Limitation and Implication for Future Research

A first limitation that warrants much consideration is the number of exploratory analyses conducted. We tested the effect of the intervention on cold pain tolerance, cold pain perception, heat pain threshold, cognitive inhibition and flexibility, divided attention, as well as mindfulness and anxiety measures. Had our main hypothesis been confirmed, these additional measures could have suggested potential pain interference reduction mechanisms. However, conducting that many tests increase Type-1 error risk. Improvement in cold pain perception in the conceptual group must therefore be considered with caution.

Secondly, although we did assess trait mindfulness (33), we did not assess the engagement or mindfulness state changes following the meditation interventions. It is not clear if the participant correctly learned how to practice meditation but could not practice by themselves during the assessment session or if the intervention was simply ineffective. Similarly, the conceptual intervention might have induced a mindfulness state, as informal forms of mindfulness exercises were presented. The limitation of manipulation checks regarding the interventions therefore limits the interpretation of our unexpected findings.

Finally, it should be noted that the present study was testing a short intervention paradigm on the interference produced by acute experimental pain. It is a practical solution for experimental research purposes, but the results may not translate directly into clinical implications on chronic pain states and to more complex interventions. Mindfulness interventions implemented in clinical practice typically last 4 to 8 weeks (9).

Nevertheless, the dissociation between meditation practice and conceptual learning about mindfulness is an original contribution that brings a novel perspective on mindfulness literature. Our unexpected results – a reduction in pain interference and in cold pain sensitivity in the conceptual group – could imply that that conceptual learning (and/or suggestions) may be relevant mechanisms by which mindfulness affects pain regulation. Of course, future studies should replicate the positive effects observed in the conceptual learning group to further confirm that such intervention can reliably decrease pain interference and reduce cold pain sensitivity.

Future studies should also assess the temporal dynamics of meditation-induced changes in pain. In the context of brief mindfulness intervention, it is possible that meditation produces a very transient reduction in pain during or immediately after the meditation practice, a valuable benefit for pain self-management that may not easily generalize to produce persistent clinical improvements, or that may go undetected using standard clinical assessment tools. Note that participants attending Mindfulness-based Stress Reduction (MBSR) training generally fail to maintain the recommended duration of meditation practice, both during (80) and in the years following the intervention (81). This lack of regular meditation practice combined with the potentially short-lived nature of the meditation-induced pain regulation might explain why mindfulness interventions do not consistently produce sustained reductions in chronic pain symptoms (10, 11, 82). In contrast, experienced meditators show a baseline reduction in pain sensitivity even when not meditating and can also reliably produce meditative analgesia (83, 84). Thus, the temporal dynamics of pain regulation by meditation in novice meditators (such as MBSR participant) should be further investigated and compared with that of meditators of different experience level (85, 86).

## Conclusion

The present results suggest that benefits from brief mindfulness meditation training reported in previous studies may be transient, temporally dependent upon an immediate practice, and may not spontaneously generalize to a separate testing session. In contrast, the conceptual learning group, who received an educational intervention on mindfulness but did not practice any formal meditation exercise, showed a reduction in pain interference and in cold pain perception. This unexpected result should be considered with caution but may reflect improved pain regulation through explicit and/or implicit suggestions that may modify ones' reactions to pain. Future studies must be conducted to delineate the relative contribution of implicit and explicit mindfulness suggestions on the reduction of acute and chronic pain symptoms.

## Data Availability Statement

The datasets presented in this article are not readily available because Data sharing with other researchers was not included in the consent form. Requests to access the datasets should be directed to Louis-Nascan Gill, gill.louis-nascan@courrier.uqam.ca.

## Ethics Statement

The studies involving human participants were reviewed and approved by Comité d'éthique de la recherche du Centre de Recherche de l'Institut Universitaire de Gériatrie de Montréal. The patients/participants provided their written informed consent to participate in this study.

## Author Contributions

L-NG, MR, VTay, KM, and PR contributed to the conception and design of the study. MR, VTab, and LB conceived the pain interference task. LB and ML developed and validated the computerized neuropsychological tests. L-NG designed and administered the mindfulness interventions, recruited participants, and assessed eligibility. KM acquired the pre- and post-intervention data. L-NG, MR, KM, and PR contributed to the analysis design and interpretation of results. L-NG prepared the manuscript with PR. This research was conducted as part of the research requirement for the Master in Psychology of L-NG. All authors contributed to the article and approved the submitted version.

## Conflict of Interest

The authors declare that the research was conducted in the absence of any commercial or financial relationships that could be construed as a potential conflict of interest. The handling Editor declared a shared secondary affiliation, though no collaboration, with two of the authors, VTab and MR.
