# Supplementary material for: Effects of Brief Mindfulness Interventions on the Interference Induced by Experimental Heat Pain on Cognition in Healthy Individuals
Source: Front Pain Res (Lausanne). 2021 Jun 2;2:673027. doi: 10.3389/fpain.2021.673027 (PMC8915756; doi:10.3389/fpain.2021.673027)
Supplement: Supplementary file 1 [file Table_1.DOCX]

Supplementary Material

# Meditation Instructions

Instructions were given in French. An English translation is reported.

## Meditation intervention

### Preparation instruction

(repeated at the beginning of every session)

Close your eyes, try to relax, and listen attentively to the instructions.

From now on, I invite you to remain still and to keep your eyes closed until I tell you to reopen them. The meditation will begin shortly. This task requires your full attention. Right now, you might be worried or agitated. Maybe you are thinking about events that happened today or this week, or perhaps you are preoccupied with future concerns.

For the next minutes, you may let go of your responsibilities, of the tasks you might have on your mind and of all worries. Imagine your thoughts about the past or the future as a big heavy bag you can now let on the floor. This big bag of yours will still be there to pick up after our session if you feel like you still need it. For now, you don't have to carry that weight. Go on, take a few moments to picture yourself putting a bag on the floor.

During the exercise, we will use the breath/of the body/everything present in our consciousness (*depending on the meditation session*) to anchor our attention to the present moment. However, there is no specific state or goal to reach. We will simply try to explore the present moment, to observe our mind as it is. Therefore, I invite you to adopt an open and curious stance, as if you were a child discovering a new game.

Before we start, make sure that your back is straight and your head aligned, but make sure you are sitting comfortably. Our posture should embody our intention to be alert but should not generate tension or discomfort. During the exercise, move slowly and mindfully if you have to.

Take three deep breaths.

### Instruction for Session One and Two

While you keep breathing deeply, direct your attention to the sensation of your breath. Maybe you can feel air entering or exiting your nose or mouth, or perhaps you notice the movement of your belly, rising as the air enters, falling as air leaves your lungs.

Now, let your respiration flow to its natural rhythm. Feeling the air going in and out of your body, feeling the belly's movement, moving with the natural rhythm of our breathing. Noticing the breath, as it naturally occurs. There is no effort to make: the body knows how and when to draw air in and out, without you needing to intervene.

(…)

Moment after moment, we simply observe the breath. We try to know, at each moment, if we are breathing in, breathing out, or maybe pausing in between the in-breath and the out-breath. We do that by trying to feel every breath. Moment after moment, we observe with curiosity and vigilance. We focus all of our attention on a body region where we feel our breath, such as our nostrils, our upper lips, our belly…

And when our attention wanders away, we gently bring it back to the breath.

Our breath may be deep and easy to perceive, or it may be shallow and hard to perceive. It does not matter how it appears. We do our best to feel it, to observe it. To do this, we are alert and focused, but it is equally essential that we remain relaxed, open and curious.

*— 2 : 00 min pause*

As we try to remain vigilant and attentive to our breath, our attention may wander. We may realize that we are distracted, that our awareness is directed toward a memory, a thought, an emotion, or anything else but the breath. It might also be possible that we feel sleepy and struggle to remain awake.

None of this is a problem. When we realize that we are not observing the respiration anymore, we mentally note it and gently bring back our attention to the feeling of the breath. Losing sight of the sensation of respiration because of mental agitation or mental tiredness is typical. It is part of the exercise. There is no need to worry about it, no need to judge ourselves. It does not matter how many times we have brought your attention back to the respiration; we keep doing it with kindness toward ourselves.

We remain curious, vigilant and relaxed. We let go of the idea of doing this exercise « well ». We are not trying to go somewhere. Instead, we try to observe what is happening right now, and respiration is a tool that we use to this end.

*Silence until the end of the session*

Note: Participants were invited to share their experience with the instructor at the end of each session. This feedback phase's goal was to ensure that participants understood the exercises and to correct their interpretation if necessary.

### Instruction for Session Three and Four

Letting the breath come and go at its natural rhythm, we take a few minutes to observe it. We feel each breath in, each breath out, wherever it appears in or on the body.

Then, slowly, we try to become aware of the different sensations that we happen to feel, at this moment, in our body. How does it feel, right now, to exist in this very body?

Take a few moments to scan all your body, from head to feet. Remaining curious and vigilant, we try our best to attend to all physical sensations that appear to our consciousness. For example, we may feel the force of gravity on our body or our clothes' light pressure. Maybe we can feel some point of contact with the ground or with the cushion.

We simply observe our sensations with curiosity and alertness.

Now, directing our attention to the top of your head, we observe all sensations in the scalp region. Anything… it could be tingling, tickling, a sense of warmth, humidity, coolness, pressure, pain…

Whatever we feel, we observe. We are not looking for good or bad sensations. We may feel pleasant, unpleasant or neutral sensations, and that is okay. We try to observe it all. We try to be conscious of everything that is happening in the scalp region.

Now, moving the attention to our face, we remain curious, calm and attentive, and we take note of all the sensations we feel on this part of our body. Pleasant, neutral or unpleasant, we remain open to every physical phenomenon that appears on our face.

At a similar pace, we observe all parts of our body in this same fashion: neck, right arm, right hand, left arm, left hand, chest, belly, upper back, lower back, etc. The order is not important, but we try not to forget any part of your body.

If it is possible, we try to feel at least three sensations in any given part of the body before moving attention elsewhere. If we don't feel anything in a given region of the body, it is not a problem. We keep our attention there for feels like about 30 seconds, remaining very attentive, then we move our attention to the next part. We can also register a blank – no sensation is a form of experience too. It is perfectly fine to observe this experience of rest from sensation.

We remain calm, like a tree deploying its roots in the soil. Whatever the sensation we experience, we stay non-reactive, open, curious and attentive. We proceed at our own pace. If we do scan every part of the body, we can start again.

We try to feel all the sensations of a given region of the body, and we try to feel it as clearly as we can. For that, we are focused and alert. But we keep in mind that this is not a competition. There is no performance here, no winners or losers. Only what happens right now is relevant. Even if we are sometimes distracted, it is not a problem. With patience, curiosity and diligence, we come back to observing sensation each time our attention wanders away.

*Silence until the end of the session*

### Instruction for Session Five

Letting the breath come and go on its natural rhythm, we take a few moments to observe it. We feel each breath in, each breath out.

Then, we try to note all the physical sensations that we happened to feel in the field of awareness of our body. We scan our body, feeling any sensation that might arise in this very moment.

Remaining curious and vigilant, we are conscious: what is happening in my body right now? We might feel the force of gravity on ourselves, the light pressure of our clothes. Maybe we can feel the point of contact with the ground or the cushion.

Then, we expand our field of awareness to everything that might present itself to our consciousness. Focusing our attention on the present moment, we take note of whatever is happening right now. Maybe we are aware of a sensation, perhaps we are aware of a sound, of a mental image, of a thought, an emotion…

Every time we observe something, we mentally name it and immediately bring our attention back to the present, to the next experience that manifests itself. So, when we become aware of a thought, we might simply label it "thought." When we become aware of a sound, whether it comes from the room or our body, we might label it "sound." When we become aware of a sensation, we might label it "sensation." Maybe, at some point, we feel joy, pleasure, or maybe we feel irritation. We could label it: "Joy,” "pleasure,” "irritation."

We label our experience as it appears, without being preoccupied with finding the perfect way to describe the experience. The label is simply a tool that helps us observe our experience, to be present.

Whenever we label an experience, we take our attention to the next moment. We just become aware of something, but then, what is happening? What is happening right now? Can we label that too? Again and again, always coming back to the now.

Remaining vigilant, open and relaxed, we continuously try to observe and label what is happening at every moment.

Maybe our attention wanders away, and we forget about the exercise, about the labeling. It is not a problem: each time we realize we are distracted, we label that distraction and gently come back to the present moment. Gently and softly, but with alertness and determination, we observe and label our experience.

## Conceptual Learning Instructions

Excerpts are taken from the French version of the book by Kabat-Zinn (2009) entitled "Everywhere you go, there you are: Mindfulness meditation in everyday life." Note that, to prevent any mention of the word "mediation" and to assure a similar script for all the participants, some sections of the chapters have been left out. The general themes of each session are presented here.

### Session 1 (Foreword, Introduction, Chapter 1)

**Themes:** Introduction to mindfulness: where it comes from, how it is used in western psychology. Presentation to the concept of mind-wandering. Explanation of how mindfulness is different from mind-wandering.

**Questions for discussion throughout the session**: Did you have any idea of what mindfulness was? Does this explanation fit your expectations?
Can you relate to this idea of "not being present?" and being on automatic pilot?
Do you think you could use mindfulness in your daily life?
Do you think what we have learned today may apply to your life?

### Session 2 (Chapter 1, Chapter 2)

**Themes:** Relationship between mindfulness and nature. Problems that mind-wandering might induce in us. Mindfulness as an art of living. Metaphor: mindfulness as standing next to a river.

**Questions for discussion throughout the session**: Have you noticed if your mind has wandered since the beginning of the session? Does the idea that mindfulness could help connect with our emotions makes any sense to you? What do you think of the metaphor of the river?

### Session 3 (Chapter 3, Chapter 4)

**Themes:** The spiritual aspect of mindfulness - letting go of expectations. The importance of attention. Exploration of the work of Henry-David Thoreau and how it relates to mindfulness.

**Questions for discussion throughout the session**: We have seen that mindfulness is not "emptying our head". Then, what do we do with our thoughts and feeling that may not be pleasant?
Is it realistic to try to accept unpleasant feelings?
Why not try to make them go away?
We have heard of Thoreau, who practiced mindfulness in the woods. Is there a way we could find inspiration from his experience?

### Session 4 (Chapter 7, chapter 9)

**Themes:** More reflections from Thoreau's experience (being in nature and savoring the moment). The metaphor of Swami Satchidananda: "surfing the wave."

**Questions for discussion throughout the session**: We have now seen a lot of different perspectives on mindfulness. In your words, how would you describe mindfulness?
What do you think of the "surfing the wave" metaphor? Do you think that could be practically applied to your life?
Do you have any example of moments where you think you could put such an open attitude to use?

### Session Five (Chapter 11, Chapter 12, Chapter 14)

**Themes:** The meaning of letting go. The meaning of a non-judgmental attitude. Having faith in the present moment.

**Questions for discussion throughout the session**:
How do you think letting go relates to mindfulness?
The author argues that "letting go" does not translate to inaction. Does that make sense to you? Does it seem paradoxical?
What does it mean to have a non-judgmental attitude?

# Additional description of neurocognitive tasks

## Modified Numerical stroop

The Numerical Stroop uses digit recognition and digits set counting instead of color and word recognition. The Stroop interference effect arises in the task condition requiring to repport the number of digits presented when the digit presented is incongruent with the number of digits to be counted (e.g., there are four digits "5" displayed) (Laguë-Beauvais, Brunet, Gagnon, Lesage, & Bherer, 2013; Sedo, 2004).

The task has four conditions: (1) in **reading condition**, identical digits are presented and must be read; (2) in **counting condition**, 1 to 6 asterisks are presented and must be counted; (3) in **inhibition condition**, participants must count the digits, but the value of the digits is either equal (congruent) or unequal (incongruent) to the number of digits, and (4) in **switching condition**, the participants must read the digits (i.e. report the value of the digits) if they are presented in a squared box or otherwise count them (i.e. report the number of digits). Inhibition and switch trials in the switching condition are analyzed separately. Therefore, when analyzing the results of this task, we consider five conditions.

Principal outcome measures are inhibition costs and switching costs. The **inhibition cost** is the performance loss due to the need to inhibit automatic responses (Stroop interference; i.e. incongruent trials). It corresponds to the ratio of the difference between the mean Reaction Time (RT) of inhibition trials and counting trials over the mean RT of counting trials. **Switching cost** is a measure of mental flexibility. It represents the performance decrement due to the necessity to switch between counting and reading tasks. It is calculated with trials in the switching condition, as the difference between the mean RT of switching trials and Inhibition trials, divided by the mean RT of inhibition trials. Higher costs indicate poorer performance.

## Dual task

This task measures the capacity to execute two different visual discrimination tasks at the same time. Participants had to identify a shape by pressing on the corresponding button on the screen. With their dominant hand, they had to identify the shape of a star, a planet or a moon quarter, while with the non-dominant hand they had to identify the shape of a dog, a snake or a bird (Lussier, Gagnon, & Bherer, 2012).

There were three types of trials: single pure, single mixed and dual mixed. In **single pure trials**, participants only had to identify items on one side of the screen on each trial. **In single mixed trials**, participants still had to identify stimuli presented onto one side at a time, but items would switch from being presented on the left or right side across trials. **During dual mixed trials**, participants had to simultaneously identify items presented on both sides simultaneously and were told to give equal priority to both sides. As for the Stroop, higher reaction time represents poorer performance.

The principal outcomes are performance costs: task-set costs and dual-task costs. **The task-set cost** is the performance decrement caused by the necessity of maintaining multiple tasks in mind. It corresponds to the difference in RT between single mixed and single pure trial, divided by RT of single pure trial. The **Dual-task cost** refers to the cost of perceiving multiple stimuli and coordinating two simultaneous motor responses. It corresponds to the RT difference between dual-mixt trials and single mixt, over RT of single mixt trials. As for the Stroop, higher cost represents poorer performance.

# Supplementary Results

## Questionnaires

**Table s1** **Questionnaire results at post-test (mean with SD in parenthesis)**

| Group | Meditation | Conceptual learning | Control  (n = 15) |
| --- | --- | --- | --- |
|  | (n = 15) | (n = 15) |  |
| MAAS | 53.53 (11.84) | 59.33 (12.89) | 21.93 (14.93) |
| FMMQ - Observe | 21.67 (4.25) | 21.00 (2.83) | 21.20 (2.91) |
| FFMQ - Awarness | 22.27 (2.22) | 22.07 (3.06) | 23.93 (2.60) |
| FFMQ -Describe | 22.93 (4.01) | 23.53 (4.09) | 27.00 (2.94) |
| FFMQ - non react | 25.27 (3.75) | 26.80 (3.03) | 22.00 (3.02) |
| FFMQ - Non-judge | 22.47 (4.31) | 23.00 (3.98) | 5.60 (3.95) |
| PCS - Rumination | 7.20 (4.00) | 6.47 (4.84) | 3.27 (3.91) |
| PCS -Magnification | 2.73 (2.46) | 3.27 (2.09) | 5.60 (2.49) |
| PCS - Helplessness | 7.60 (4.76) | 6.80 (4.83) | 21.93 (4.95) |
| STAI-State | 34.60 (9.16) | 28.53 (5.88) | (29.88. 8.02) |

**Table s2 Test-retest and interaction statistics for the questionnaires.**

None of the questionnaire showed any interaction effect, showing no evidence of an effect of the intervention on mindfulness, anxiety and pain catastrophizing measures.

|  | **Test-retest correlation [r]**  ** = p < 0.05, ** = p < 0.01* | **Interaction statistics (ANOVA) [F, ƞ²]**  *No significant interaction* |
| --- | --- | --- |
| MAAS | 0.78** | 0.45, 0.04 |
| FMMQ - Observe | 0.48** | 0.15, 0.01 |
| FFMQ - Awarness | 0.44** | 0.71, 0.02 |
| FFMQ -Describe | 0.69** | 3.17, 0,13 |
| FFMQ - non react | 0.62** | 2.09, 0.09 |
| FFMQ - Non-judge | 0.77** | 0.56, 0.03 |
| PCS - Rumination | 0.61** | 0.04, 0.01 |
| PCS -Magnification | 0.48** | 1.22, 0.06 |
| PCS - Helplessness | 0.77** | 1.89, 0.08 |
| STAI-State | 0.41 | 1.32, 0.06 |

##
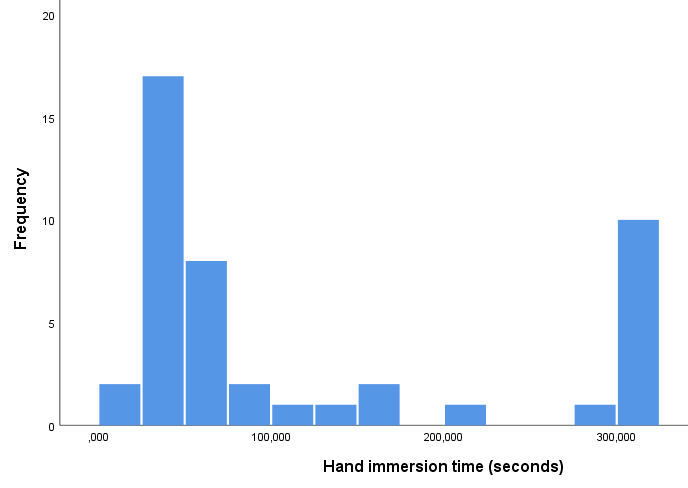
Cold pressor


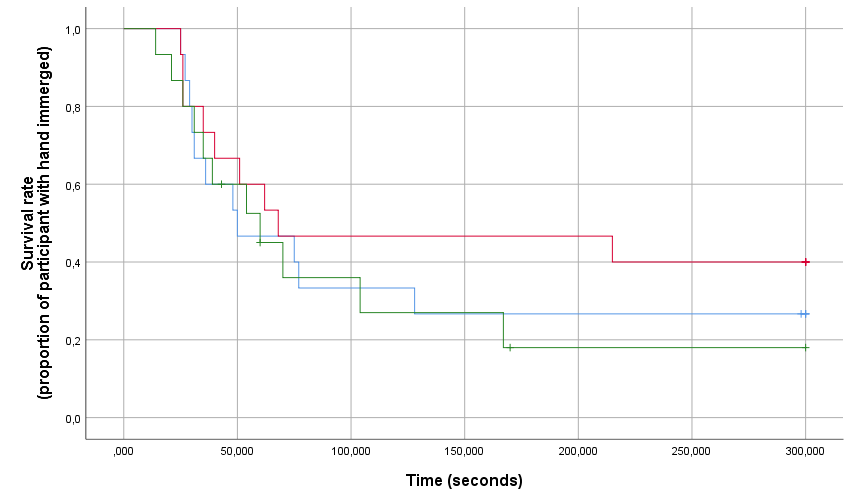
Figure S1 Frequency of the hand immersion time (in seconds). Because of the bimodal distribution, the mean immersion time (117,69 seconds) may not adequately represent the data. Given that the normality of the distribution could not be assumed, non-parametric tests were conducted on hand immersion time.

Figure S2 Survival curves at pre-test. Blue line = Meditation, red line = Conceptual control, green line = Control.

##
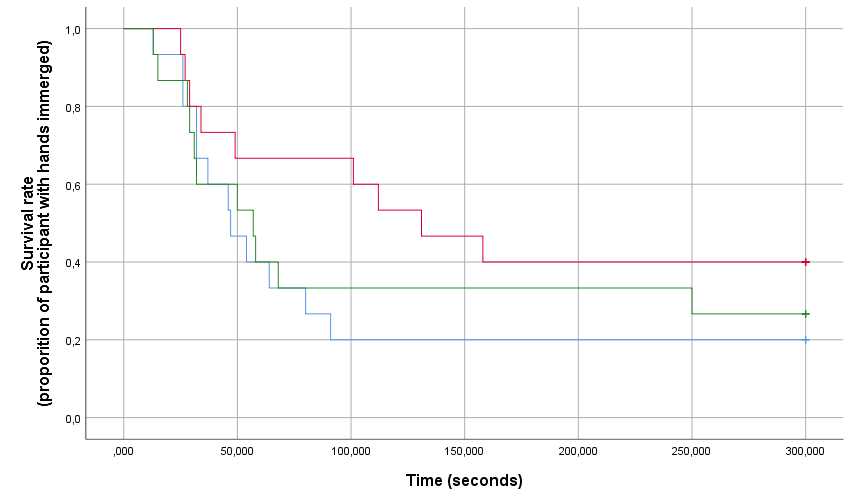


Figure S3 Survival curves at post-test. Blue line = Meditation, red line = Conceptual control, green line = Control.

## Sensory calibration

Sensory calibration was performed before the interference task to adjust the thermal stimuli individually and control for subject-related differences in sensitivity at each session. The moderately painful temperature (VAS140) and the pain threshold (VAS 100) observed in each group and in each session are reported in Table s3. There was a significant increase between session 1 and 2 for the temperature corresponding to VAS100 [Effect of SESSION: *F*(1,42) = 39.25, *p* < .001, ƞ²= 0.49] and VAS140 [*F*(1,42) = 33.76 *p* < .001, ƞ²= 0.45]. There was no main effect of GROUP [VAS100: *F*(2,42) < 1 p = .8, ƞ² = 0.02; VAS140: F (2,42) < 1, *p* = .9, ƞ² = 0.04] and no significant interaction GROUP X SESSION on either VAS100 temperature [*F*(2,42) < 1; *p* = 0.8, ƞ² = 0.01] or VAS140 temperature [*F*(2,42) < 1; *p* = .9, ƞ² = 0.01]. These results show no evidence that the intervention affected pain sensitivity.

**Table s3
Mean (95% CI) of warm temperature (VAS70), pain threshold (VAS100) and moderately painful temperature (VAS140) measured in the sensory calibration at each testing session in the three groups**

| Group | Meditation | Conceptual learning | Control  (n = 15) |
| --- | --- | --- | --- |
|  | (n = 15) | (n = 15) |  |
| VAS 70 temperature, session one | 44.77 ^o^C  (43.98 to 45.47 ^o^C) | 44.08 ^o^C  (42.88, 45.28 ^o^C) | 44.7 ^o^C  (43.76, 45.64 ^o^C) |
| VAS 70 temperature, session two | 45.36 ^o^C  (44.49 to 46.23 ^o^C) | 45.01 ^o^C  (43.85 to 46.17) | 45.63^o^C  (44.93 to 46.32 ^o^C) |
| VAS 100 temperature, session one | 46.3^o^C  (45.75 to 46.86^o^C) | 45.91^o^C  (45.05 to 46.78 ^o^C) | 46.29 ^o^C  (45.60 to 46.97) |
| VAS 100 temperature, session two | 46.87 ^o^C  (46.20 to 47.54^o^C) | 46.63 ^o^C  (45.70 to 47.57^o^C) | 46.96^o^C  (46.41 to 47.51^o^C) |
| VAS 140 temperature, session one | 47.79^o^C  (47.39 to 48.18^o^C) | 47.45 ^o^C  (46.89 to 48.02^o^C) | 47.81^o^C  (47.36 to 48.26^o^C) |
| VAS140 temperature, session two | 48.15 ^o^C  (47.74 to 48.56^o^C) | 47.90 ^o^C  (47.39 to 48.41^o^C) | 48.19 ^o^C  (47.81 to 48.57^o^C) |

## Two-back calibration

The mask duration in the 2-back task was adjusted individually before the interference task to control for individual baseline differences in performance at each session. Group mean and CI are reported for each session in Table s4. Mask duration was significantly longer in session 2, consistent with an improved performance [main effect of SESSION: *F*(1, 41) =49.76, *p* < .001, ƞ² = 0.49]. A main effect of group GROUP was revealed [*F*(2, 41) =4.27, *p* = .023, ƞ² = 0.44], with the mask duration being significantly shorter in the Conceptual than the Control group. There was no group GROUP x X session SESSION interaction [*F*(2, 41) =0.49, p = .9, ƞ² <0.01], showing no evidence that the interventions affected the 2-back performance.

**Table s4**

**Mean (95% CI) mask duration after calibration and 2-back performance (A statistic) during interference task each testing session in the three groups**

| Group | Meditation | Conceptual learning | Control |
| --- | --- | --- | --- |
|  | (n = 14) | (n = 15) | (n = 15) |
| Mask duration session 1 (ms) | 378  (222 to 535) | 264  (215 to 334) | 533  (368 to 698) |
| Mask duration session 2 (ms) | 553  (417 to 689) | 454  (358 to 549) | 693  (501 to 884) |
| 2 back performance during VAS70 stimulation, session 1 | .882  (.843 to .921) | .870  (.832 to .903) | .846  (.794 to .897) |
| 2 back performance during VAS70 stimulation, session 2 | .879  (.840 to .919) | .835  (.799 to .870) | .847  (.794 to .9) |
| 2 back performance during VAS140 stimulation, session 1 | .856  (.814 to .898) | .803  (.754 to .852) | .814  (.773 to .854) |
| 2 back performance during VAS140 stimulation, session 2 | .850  (.812 to .887) | .833  (.781 to .885) | .785  (.739 to .852) |

## Stroop

Means and CI of RT correct responses for each Stroop conditions and for inhibition and switching costs are presented in Table S5. Repeated measure ANOVA on Stroop session RTs revealed a significant difference between conditions [*F*(1.75,76.77) = 243.07, *p* > .001, ƞ² = 0.85], while post-hoc analysis showed a gradual and significant (*p* > .01) increase from condition one to five (i.e., RTs were increasing with condition difficulty). These results confirmed that the Stroop task worked as expected.

The analysis revealed faster RTs in sessions 2 [main effect of session: *F*(1, 41) = 168.08, *p* < .001, ƞ² = 0.8]. However, some condition showed larger improvements between sessions [condition x session interaction *F*(2.189, 164) = 27.7, p < .001 ƞ² = 0.40]. A main effect of condition was also revealed [F(1.84,78.76) = 310.01, p < 0.001, ƞ² = 0.88], but there was no GROUP x CONDITION interaction [*F*(3.8,78.76) = 1.45, *p* = 0.2, ƞ² = 0.07], no significant GROUP x SESSION interaction [*F*(2,41) = 1.49, *p* = .24, ƞ² = 0.07] and no GROUP x SESSION x CONDITION interaction [*F*(4.379, 164) = 1.56, *p* = 0.19, ƞ² = 0.07]. This indicates an absence of effect of the interventions on mean RTs.

More detailed analyses of inhibition costs revealed no significant main effect or interaction [GROUP: *F*(2,41) = 0.5, *p* = 0.6; SESSION: F(1,41) = 1.065, p = 0.30; INTERACTION: F(2,41) = 0,665, *p* = .52]. For the switching cost, analysis showed no main effect of group [*F*(2,41) = 0.241, *p* = .78, ƞ² = 0.011], but revealed a near significant increase in cost between sessions [F(1,41) = 4.018, p = 0.051, ƞ² = 0.087]. No interaction effect was revealed [F(2,41) = 0.5, *p* = .7]. These results indicate that the interventions did not affect Stroop performance in general nor inhibition and switching costs. Overall, there was no indication of a differential effect of the interventions on Stroop performance.

| Table S5 | | | | |
| --- | --- | --- | --- | --- |
| *Mean (SD) reaction time (in ms) in the various conditions of the Stroop task and cost analyses for inhibition and switching for the two testing sessions in each group* | | | | |
| Group | Meditation | Concept. learning | Control |  |
|  | (n = 15) | (n = 15) | (n = 14) |  |
| Reading (condition 1) RT, session 1 | 822  (765 to 880) | 791  (755 to 829) | 875  (812 to 938) |  |
| Reading (condition 1) RT, session 2 | 776  (726 to 828) | 746  712 to 780 | 854  (785 to 922) |  |
| Counting (condition 2) RT, session 1 | 851  (799 to 903) | 808  (769 to 846) | 916  (854 to 977) |  |
| Counting (condition 2) RT, session 2 | 834  (784 to 885) | 749  727 to 772 | 808  (755 to 861) |  |
| Inhibition (condition 3) RT, Session 1 | 949  (905 to 993) | 916  (854 to 977) | 1042  (985 to 1098) |  |
| Inhibition (condition 3) RT Session 2 | 909  (843 to 755) | 839  (793 to 886) | 945  (882 to 1008) |  |
| Inhibition trials in switching condition (condition 4) RT, session 1. | 1201  (1106 to 1296) | 1030  (953 to 1106) | 1089  (1036 to 1142) |  |
| Inhibition trials in switching condition (condition 4) RT, session 2 | 953  (894 to 1011) | 906  (849 to 962) | 1028  (965 to 1092 |  |
| Switch trials RT in switching condition (condition 5), session 1 | 1023  (960 to 1078) | 982  (920 to 1044) | 1100  (1028 to 1172) |  |
| Switch trials in switching condition (condition 5) RT, session 1 | 1024  (960 to 1087) | 982  (920 to 1044) | 1101  (1029 to 1172) |  |
| Inhibition cost, session 1` | .12  (.07 to 0.17) | .13  .08 to 0.18 | .16  (.11 to .21) |  |
| Inhibition cost, session 2 | .12  (.09 to .16) | .12  (.07 to .17) | .14  (.80 to .19) |  |
| Switching cost session 1 | .15  (.12 to .20) | .18  (.11 to .24) | .16  (.11 to.23) |  |
| Swtiching cost session 2 | .19  (.16 to .23) | .22  (.17 to .27) | .18  (.12 to .23) |  |

## Dual-task

Mean RT of correct responses and CI in the different dual-task conditions and task set cost and dual task cost are presented in table S6. Repeated measures ANOVA on baseline dual-task performance revealed a significant RT difference between conditions [*F*(1.24, 54.38) = 798.62, *p* > .001, ƞ² = 0.95]. Higher RTs were observed for more demanding conditions, with a gradual and significant (p > .001) increase from condition one to tree. These effects indicate that the dual-task worked as intended.

The analysis revealed faster RTs in sessions 2 [*F*(1, 42) = 10.51, *p* = .002, ƞ² = 0.2]. Overall RTs were significantly different between groups [ *F*(2, 42) = 4.57, *p* = 0.016, ƞ² = 0.18], with the control group having slightly longer RTs then the conceptual learning group (*p* < 0.05). No SESSION x GROUP interaction was observed [*F*(2, 42) = 1.76, p =  .18, ƞ² = 0.08]. Analysis also revealed a significant main effect of condition [*F*(1.16, 48.5) = 999.45, *p* < .001, ƞ² = 0.96] as well as a CONDITION x SESSION interaction [*F*(1.614 48.5) = 4.69, *p* = .018, ƞ² = 0.1]. However, there was no significant GROUP X SESSION x CONDITION interaction [F(3.23, 84) = 2.06, *p* = .11, ƞ² = 0.06]. This indicates that the reduction in RTs in session 2 did not vary significantly between groups.

Analyses of task set cost indicated that the decrease in session two did not reach statistical significance [main effect of SESSION: *F*(1,42) = 3.54, *p* = .78, ƞ² = 0.08]. A main effect of GROUP was found [F(2,42) = 3.32, p = .46, ƞ² = 0.14], with the meditation group having slightly higher cost then the conceptual learning group (p < 0.05). No significant GROUP x SESSION interaction [*F*(2, 42) = 1.51, *p* = .24, ƞ² = 0.07] was found. These effects reveal no evidence that the intervention changed task set cost.

Analyses of the dual-task cost revealed a diminution in dual task cost between session one and two [main effect of SESSION: F(1,42) = 492,89 p < 0.001, ƞ² = 0.92], but revealed no main effect of GROUP [*F*(2,42) = 0.93, *p* = 0.4, ƞ² = 0.04], nor any GROUP x SESSION interaction [*F*(2,42) = 0.62, *p* = .5, ƞ² = 0.03 ], showing no evidence that the intervention had any effect on dual task cost.

| Table S6 | | | | |
| --- | --- | --- | --- | --- |
| *Dual-task results. RT are in milliseconds (mean with standard deviations in parentheses)* | | | | |
| Group | Meditation | Conceptual learning | | Control |
|  | (n = 15) | (n = 15) | | (n = 15) |
| Single pure (condition 1) RT, session 1 | 697  (656 to 739) | 675  (650 to 699) | 742  (697 to 786) | |
| Single Pure RT (condition 1) RT, session 2 | 681  (655 to 706) | 667  (637 to 698) | 722  (678 to 765) | |
| Single Mixt RT (condition 2) RT, session 1 | 881  (812 to 950) | 789  (743 to 836) | 895  (823 to 967) | |
| Single Mixt RT(condition 2) RT, session 2 | 819  (777 to 861) | 779  (728 to 831) | 857  (809 to 905) | |
| Dual Mixt (condition 3) RT, session 1 | 1376  (1280 to 1472) | 1230  (1137 to 1331) | 1430  (1323 to 1537) | |
| Dual Mixt (condition 3) RT, session 2 | 1282  (1198 to 1367) | 1219  (1106 to 1333) | 1395  (1306 to 1483) | |
| Task set cost, session 1 | .260  (.225 to .295) | .170  (.120 to .219) | .206  (.149 to .262) | |
| Task set cost, session 2 | .203  (.163 to .244) | .167  (.125 to .208) | .190  (.149 to .232) | |
| Dual task cost, session 1 | .567  (.510 to .624) | .560  (.494 to .626) | .602  (.558 to .646) | |
| Dual task cost session 2 | .564  (.514 to .612) | .559  (.497 to .620) | .627  (.573 to .682) | |

# References

Laguë-Beauvais, M., Brunet, J., Gagnon, L., Lesage, F., & Bherer, L. (2013). A fNIRS investigation of switching and inhibition during the modified Stroop task in younger and older adults. *NeuroImage, 64*, 485-495. Retrieved from <http://www.sciencedirect.com/science/article/pii/S1053811912009524>. doi:<https://doi.org/10.1016/j.neuroimage.2012.09.042>

Lussier, M., Gagnon, C., & Bherer, L. (2012). An investigation of response and stimulus modality transfer effects after dual-task training in younger and older. *Frontiers in Human Neuroscience, 6*.

Sedo, M. A. (2004). ['5 digit test': a multilinguistic non-reading alternative to the Stroop test]. *Rev Neurol, 38*(9), 824-828.
